# Clinical Usefulness of Response Profiles to Rapidly Incremental Cardiopulmonary Exercise Testing

**DOI:** 10.1155/2013/359021

**Published:** 2013-05-12

**Authors:** Roberta P. Ramos, Maria Clara N. Alencar, Erika Treptow, Flávio Arbex, Eloara M. V. Ferreira, J. Alberto Neder

**Affiliations:** ^1^Pulmonary Function and Clinical Exercise Physiology Unit (SEFICE), Division of Respiratory Diseases, Department of Medicine, Federal University of Sao Paulo (UNIFESP), Rua Francisco de Castro 54, Vila Mariana, 04020-050 São Paulo, SP, Brazil; ^2^Division of Respiratory and Critical Care Medicine, Department of Medicine, Queen's University and Kingston General Hospital, Richardson House, 102 Stuart Street, Kingston, ON, Canada K7L 2V6

## Abstract

The advent of microprocessed “metabolic carts” and rapidly incremental protocols greatly expanded the clinical applications of cardiopulmonary exercise testing (CPET). The response normalcy to CPET is more commonly appreciated at discrete time points, for example, at the estimated lactate threshold and at peak exercise. Analysis of the response profiles of cardiopulmonary responses at submaximal exercise and recovery, however, might show abnormal physiologic functioning which would not be otherwise unraveled. Although this approach has long been advocated as a key element of the investigational strategy, it remains largely neglected in practice. The purpose of this paper, therefore, is to highlight the usefulness of selected submaximal metabolic, ventilatory, and cardiovascular variables in different clinical scenarios and patient populations. Special care is taken to physiologically justify their use to answer pertinent clinical questions and to the technical aspects that should be observed to improve responses' reproducibility and reliability. The most recent evidence in favor of (and against) these variables for diagnosis, impairment evaluation, and prognosis in systemic diseases is also critically discussed.

## 1. Introduction

Cardiopulmonary exercise testing (CPET) provides a means of unraveling abnormal physiologic functioning which may not be apparent at rest [1, 2]. The advent of microprocessed CPET systems [3] increased our technical capabilities in recording several variables throughout a single exercise bout—even of a relatively “short” duration of 10 minutes [4, 5]. The response normalcy to rapidly incremental CPET is more commonly judged by comparing the observed values at discrete time points (e.g., at the estimated lactate threshold (LT) and at peak exercise) with those previously obtained in apparently healthy subjects [6, 7]. It should be noted, however, that relying only in such discrete analysis leads to substantial loss of physiologic information given by the observation of the responses profiles during submaximal exercise and recovery [8–11].

In this context, authoritative textbooks [2, 12] and guidelines [13, 14] advocated that the trending of certain variables is a crucial component of the interpretative strategy as they might show substantial abnormalities even when the discrete values are still within the expected range [15–17]. Moreover, the response dynamics are highly reproducible [8–11], encompassing a range of exercise intensities which are likely to be faced by the patients in daily life [18–26]. Although the scientific foundations supporting their use have long been established, [8–17] they are still not routinely assessed and clinically valued in practice.

The purpose of this brief review, therefore, is to emphasize the practical usefulness of analyzing the response profiles of selected variables during rapidly-incremental CPET. Special care is taken to physiologically justify their use to answer relevant clinical questions and to the technical details that should be observed to improve responses' reproducibility and reliability. The response profiles to be discussed, however, are applicable to ramp-incremental [4] cycle ergometry, and the practitioner should be aware that different patterns of response can be anticipated if other ergometers (e.g., treadmill) and protocols (e.g., step-like) are used. 

## 2. Metabolic Responses

### 2.1. Estimated Lactate Threshold

#### 2.1.1. Physiological Background

The rate at which arterial lactate anions [Lac^−^]_a_ and the associated proton (H^+^) accumulate as exercise progresses is directly related to the ratio between lactic acid (LA) release as a final byproduct of muscle anaerobic glycolysis and LA clearance by metabolism and buffering [29–31]. Although there seems to exist a period of time—not a discrete time point—in which LA production exceeds its rate of clearance, the term LA “threshold” (LT) [32, 33] is widely used. LA production increases as tissue O_2_ delivery diminishes [34] though some LA can be produced without any evidence of tissue hypoxia [35]. This justifies the notion that LA release during exercise is a reasonably sensitive (albeit non-specific) [36] marker of tissue anaerobiosis.

LA dissociates fast in Lac^−^ and H^+^ in the physiological pH; that is, it is a strong acid. Plasma bicarbonate (HCO_3_^−^) is the main buffer of lactic acidosis leading to the formation of carbonic acid (H_2_CO_3_) which in turn dissociates into carbon dioxide (CO_2_) and water; that is,

(1) $$\begin{array}{c} {\text{H}}^{+}{\text{Lac}}^{-}+{{\text{HCO}}_{3}}^{-}\Leftrightarrow {\text{H}}_{2}{\text{CO}}_{3}\Leftrightarrow {\text{CO}}_{2}+{\text{H}}_{2}\text{O}. \end{array}$$

Although this reaction has the advantage to turn a fixed acid into a volatile gas, the “extra-CO_2_” (approximately 22–26 mL of additional CO_2_ is produced from each mEq decrease of [HCO_3_^−^]) [31] derived from buffering of Lac^−^-associated protons will not only accelerate CO_2_ output $\left({\dot{V}}_{{\text{CO}}_{2}}\right)$ relative to O_2_ uptake $\left({\dot{V}}_{{\text{O}}_{2}}\right)$ but also stimulate ventilation $\left({\dot{V}}_{\text{E}}\right)$. These phenomena underlie the techniques for a noninvasive estimation of the LT.

#### 2.1.2. Technical Considerations

**Figure 1:**
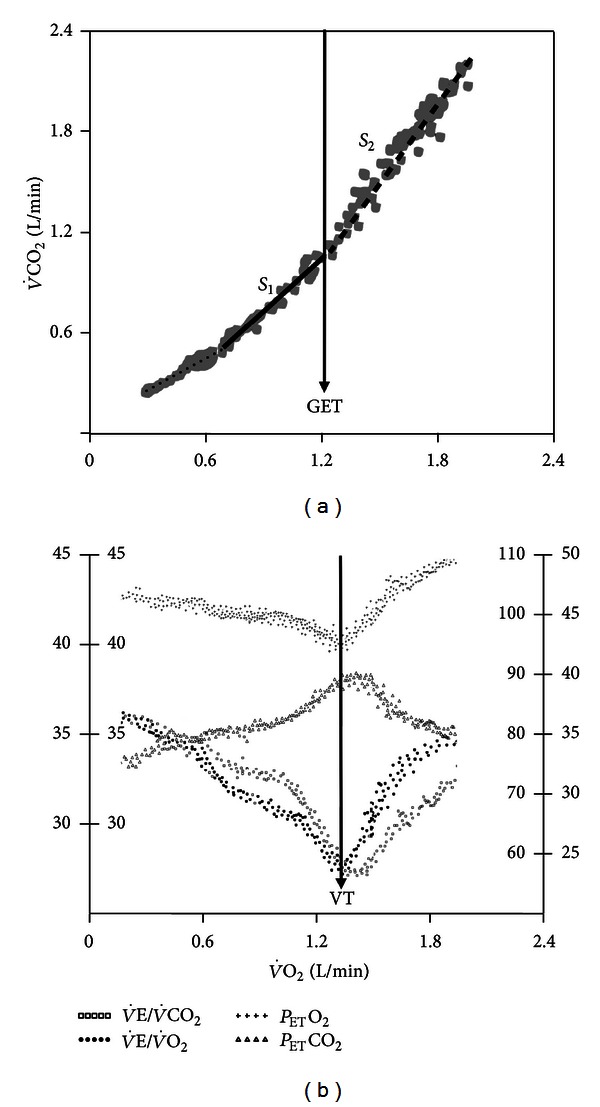
Noninvasive estimation of the lactate threshold by the *V*-slope method (gas exchange threshold (GET), *panel* (a)) and the ventilatory method (ventilatory threshold (VT), *panel* (b)) in a normal subject. Note that the GET slightly precedes the VT as the later depends on the ventilatory response to the “extra-CO_2_” generated by buffering of H^+^ associated with (lactate) increase. *S*_1_ and *S*_2_ refer to the two sequential slopes (before and after the GET) with *S*_2_ being characteristically steeper than *S*_1_ (i.e., slope inclination >1.)

As LA is buffered by HCO_3_^−^, ${\dot{V}}_{{\text{CO}}_{2}}$ increases (1) out of proportion of ${\dot{V}}_{{\text{O}}_{2}}$, and a plot between these variables will show a discernible breakpoint; that is, the ${\dot{V}}_{\text{C}{\text{O}}_{2}}$-${\dot{V}}_{{\text{O}}_{2}}$ relationship evidences an increased slope at the point of [Lac^−^]_a_ increase. This is more commonly referred as the *gas exchange threshold *and determined by the *V-slope* method (Figure 1(a)) [37]. Increase in ${\dot{V}}_{\text{C}{\text{O}}_{2}}$ will drive ${\dot{V}}_{\text{E}}$ in its direct proportion leading the latter to increase faster than ${\dot{V}}_{{\text{O}}_{2}}$. The consequent increase in ${\dot{V}}_{\text{E}}/{\dot{V}}_{{\text{O}}_{2}}$ (and the end-tidal partial pressure for O_2_, *P*_ET_O_2_) with a stable ${\dot{V}}_{\text{E}}/{\dot{V}}_{\text{C}{\text{O}}_{2}}$ (and *P*_ET_CO_2_) establishes the so-called *ventilatory threshold *(Figure 1(b)) [38]. It should be noted that despite reflecting the same phenomenon (LA buffering), the *gas exchange threshold* slightly precedes the *ventilatory threshold* (VT) (Figure 1). After the LT, ${\dot{V}}_{\text{E}}/{\dot{V}}_{\text{C}{\text{O}}_{2}}$ and *P*_ET_  CO_2_ remain stable for a variable period of time during the “isocapnic buffering”. However, as more H^+^ is released with further increases in work rate,$\;{\dot{V}}_{\text{E}}$ eventually increases out of proportion to ${\dot{V}}_{\text{C}{\text{O}}_{2}}$ at the respiratory compensation point (RCP) thereby leading to alveolar hyperventilation and progressive reductions in *P*_ET_  CO_2_ towards the end of the test (Figure 1(b)).

Irrespective of the denomination, the following technical aspects for the LT estimation should be noted:

1. automatic estimations (by the CPET software) should be viewed with caution and routinely double-checked with manually determined values;
2. if an unitary tangent is used to estimate the LT in the *V*-slope plot, the range of ${\dot{V}}_{{\text{O}}_{2}}$ and ${\dot{V}}_{\text{C}{\text{O}}_{2}}$ values should be the same as any discrepancy would invalidate its underlying mathematical (and physiological) principles [37] (Figure 1(b));
3. use of discrete R (${\dot{V}}_{\text{C}{\text{O}}_{2}}/{\dot{V}}_{{\text{O}}_{2}}$) values (i.e., > 1 from tabular data) as indicative of the LT might lead to erroneous estimations;
4. ${\dot{V}}_{{\text{O}}_{2}}$ at any particular WR during a ramp-incremental test is lower than the steady-state ${\dot{V}}_{{\text{O}}_{2}}$ value at that same WR due to a variable ${\dot{V}}_{{\text{O}}_{2}}$ kinetics delay. As a result, the WR corresponding to ${\dot{V}}_{{\text{O}}_{2}}$ LT precedes the WR in which the LT was identified by approximately 30–45 s (or even more in patients) [4]. Accordingly, if one is interested in exercising a subject at the ${\dot{V}}_{{\text{O}}_{2}}$ LT, the selected WR should lead the WR-LT by this timeframe;
5. a given change in ${\dot{V}}_{\text{E}}$ has a greater effect on CO_2_ release than O_2_ uptake by the lungs; consequently, preexercise hyperventilation may deplete the amount of CO_2_ stored in the body without major effects on O_2_ stores [39]. As the body capacitance for CO_2_ increases during the early phase of the ramp, repletion of the CO_2_ stores slows ${\dot{V}}_{\text{C}{\text{O}}_{2}}$ relative to ${\dot{V}}_{{\text{O}}_{2}}$; that is, ${\dot{V}}_{\text{C}{\text{O}}_{2}}$-${\dot{V}}_{{\text{O}}_{2}}$ slope in this region becomes shallow (“*S*_1_” in Figure 1(a)). As the body CO_2_ reservoirs are filled in with exercise progression, the rate of CO_2_ storage will decrease thereby accelerating ${\dot{V}}_{\text{C}{\text{O}}_{2}}$ relative to ${\dot{V}}_{\text{C}{\text{O}}_{2}}$ [40]. This might mistakenly suggest the onset of lactic acidosis, that is, a “pseudo-LT” [41]. Precautions should therefore be taken to avoid hyperventilation prior to the noninvasive estimation of LT by the *V*-slope method;
6. LT should always be expressed relative to *predicted* ${\dot{V}}_{{\text{O}}_{2}}$ peak not to the *attained* ${\dot{V}}_{{\text{O}}_{2}}$ peak, especially in patient populations where the latter procedure might create a false concept of preserved (or even increased) ${\dot{V}}_{{\text{O}}_{2}}$ LT, and
7. ${\dot{V}}_{{\text{O}}_{2}}$ peak declines with senescence at a steeper rate than ${\dot{V}}_{{\text{O}}_{2}}$ LT; that is, ${\dot{V}}_{{\text{O}}_{2}}$ LT (% ${\dot{V}}_{{\text{O}}_{2}}$ peak) *increases* as a function of age in both genders [41–43].

#### 2.1.3. Clinical Usefulness

The physiologic changes associated with [Lac^−^]_a_ and H^+^ accumulation (e.g., metabolic acidosis, impaired muscle contraction, hyperventilation, and altered ${\dot{V}}_{{\text{O}}_{2}}$ kinetics) are important to document clinically as they are associated with reduced cardiopulmonary performance. An early LT is a marker of impaired aerobic metabolism [44–49] due to insufficient O_2_ delivery, increased recruitment of fast-twitch type II fibers which are metabolically less efficient than the slow-twitch type I fibers (i.e., have a greater O_2_/ATP ratio), and/or mitochondrial enzymatic dysfunction. The isolated analysis of the LT does not allow the differentiation of cardiovascular limitation from sedentarity though a severely decreased LT (e.g., <40% predicted ${\dot{V}}_{{\text{O}}_{2}}$ peak) [6] is more frequently found in patients. A low LT has been found useful to predict an increased risk of post-operatory complications in the elderly [50, 51], worse prognosis in chronic heart failure (CHF) [52], and disease severity in pulmonary arterial hypertension (PAH) [53]. On the other hand, improvements in LT after pharmacological and nonpharmacological interventions have been associated with increased functional performance in a range of clinical populations [54–69]. Although there is lack of evidence that training at (or above) the ${\dot{V}}_{{\text{O}}_{2}}$ LT is essential to improve exercise capacity in patients with CHF, coronary artery disease (CAD), and chronic obstructive pulmonary disease (COPD), training at higher intensities elicits larger physiological adaptations in less severe patients who are able to tolerate such regimens [54, 70, 71]. Training at the ${\dot{V}}_{{\text{O}}_{2}}$ LT also seems to reduce the risk of complications during early phases of cardiac rehabilitation [72, 73]. In patients with COPD, however, LT cannot always be identified (even using the *V-slope* method), and when identified it varies widely as expressed in ${\dot{V}}_{{\text{O}}_{2}}$% peak [74]. In fact, important subjective improvements after rehabilitation can be found despite the lack of measurable physiological effects [75] which casts doubt on its usefulness to target exercise training intensity in these patients.

### 2.2. Δ Oxygen Uptake $({\dot{V}}_{{\text{O}}_{2}})/\Delta$ Work Rate (WR)

#### 2.2.1. Physiological Background

From a relatively constant value of 500 mL/min at unloaded pedaling, ${\dot{V}}_{{\text{O}}_{2}}$ increases linearly as exercise progresses during a rapidly-incremental exercise test [4]. The slope of the $\Delta {\dot{V}}_{{\text{O}}_{2}}/\Delta \text{WR}$ relationship, therefore, is an index of the overall gain of the ${\dot{V}}_{{\text{O}}_{2}}$ response, and normal values would indicate adequate metabolic cost for the production of a given power output [4, 8].

#### 2.2.2. Technical Considerations

**Figure 2:**
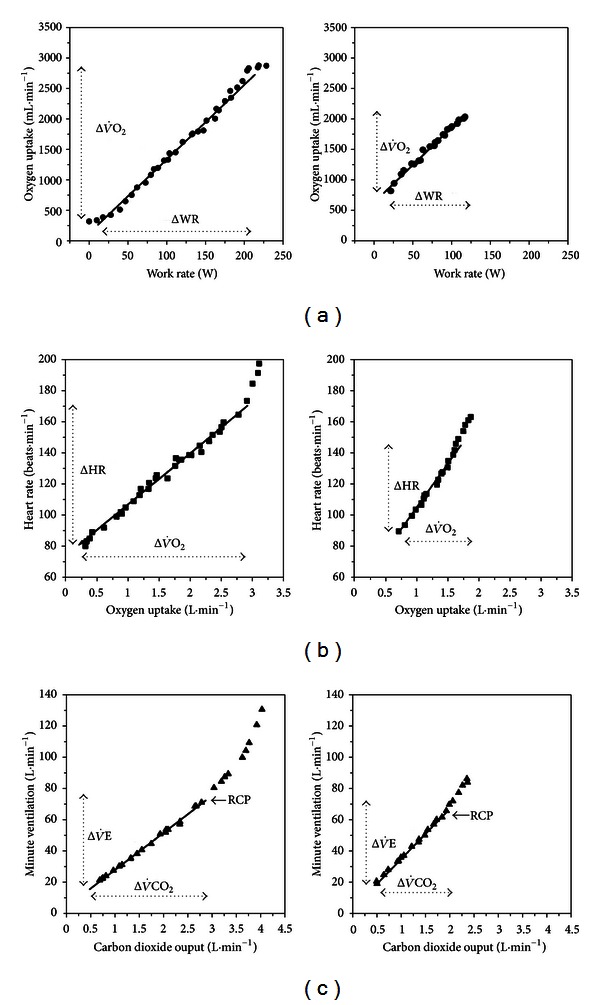
Procedures to establish 3 dynamic submaximal relationships by simple linear regression during incremental CPET in young (24-yr-old, left panels) and old (70-yr-old, right panels) subjects. (a) Δ oxygen uptake $({\dot{V}}_{{\text{O}}_{2}})/\Delta$ work rate (WR); (b) Δ heart rate$/\Delta {\dot{V}}_{{\text{O}}_{2}}$ (c) Δ minute ventilation $({\dot{V}}_{\text{E}})/\Delta$ carbon dioxide output $\left({\dot{V}}_{{\text{CO}}_{2}}\right)$. The arrows show the range of values considered for analysis. RCP is the respiratory compensation point. *(Modified with permission from [10].) *

For an accurate calculation of the $\Delta {\dot{V}}_{{\text{O}}_{2}}/\Delta \text{WR}$ slope, any delay in ${\dot{V}}_{{\text{O}}_{2}}$ increase at the start of the ramp or any eventual plateau near the end of exercise should be discarded (Figures 2(a) and 4). Considering that the LT can potentially distort the response's linearity [157–160], it is advisable to check if there is an inflection point in the $\Delta {\dot{V}}_{{\text{O}}_{2}}/\Delta \text{WR}\;$at the LT. If this is discernible, the slope should be calculated over the sub-LT range.

#### 2.2.3. Interpretative Issues

**Figure 3:**
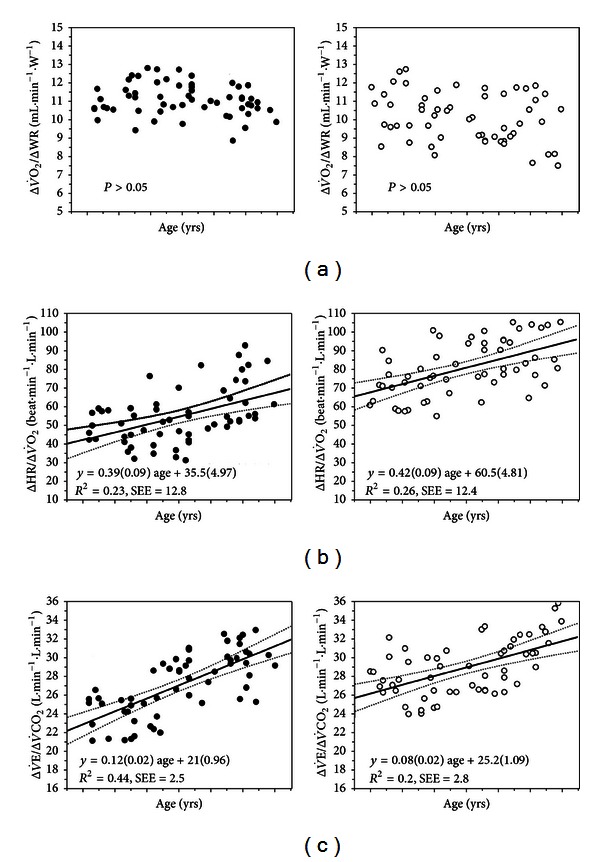
The submaximal relationships depicted in Figure 2 as a function of age in males (left panels) and females (right panels). Regression lines are shown with their respective 95% confidence intervals for those relationships in which the variables were influenced by age. Regression coefficients and intercepts of the linear prediction equations are depicted with their respective standard error of the estimate (SEE). *(Modified with permission from [10]). *

**Figure 4:**
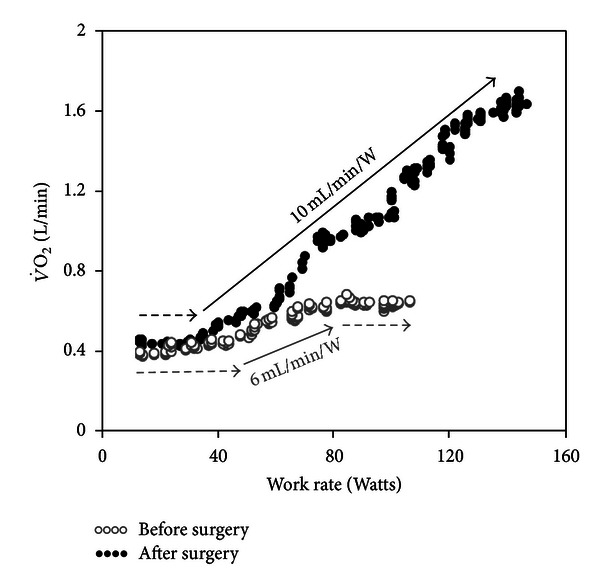
Oxygen uptake $({\dot{V}}_{{\text{O}}_{2}})/$work rate (WR) relationship during ramp-incremental CPET before and after pulmonary endarterectomy in a 21-year-old male with thromboembolic occlusion of the left pulmonary artery. Note that after the surgery, peak ${\dot{V}}_{{\text{O}}_{2}}$ increased not only due to a higher peak WR but also owing to a large improvement in $\Delta {\dot{V}}_{{\text{O}}_{2}}/\Delta \text{WR}$.

$\Delta {\dot{V}}_{{\text{O}}_{2}}/\Delta \text{WR}$ is not significantly influenced by the training status, ageing, or gender (Figure 3(a)) [2, 10, 12–14]. A shallow $\Delta {\dot{V}}_{{\text{O}}_{2}}/\Delta \text{WR}$ over the entire range of values and/or a shift from a linearly increasing profile to a shallower rate of change has been shown to be indicative of circulatory dysfunction [77–80] (Figure 4) and severe impairment in mitochondrial function [81]. The latter pattern of response has been found to enhance ECG sensitivity to detect myocardial ischemia [82–86], and some studies suggested that it might be useful to unravel early abnormalities in the coronary microcirculation [87, 88].

### 2.3. ${\dot{V}}_{{\text{O}}_{2}}$ Efficiency

#### 2.3.1. Physiological Background

${\dot{V}}_{\text{E}}$ increases curvilinearly relative to ${\dot{V}}_{{\text{O}}_{2}}$ in response to a ramp-incremental exercise test. At least in theoretical grounds, several variables known to interfere with both $\;{\dot{V}}_{\text{E}}$ and ${\dot{V}}_{{\text{O}}_{2}}$ would bear an influence in this relationship; that is, it is deemed to be modulated by cardiovascular, pulmonary, and muscular factors [161–168]. Most authors have expressed the $\;{\dot{V}}_{\text{E}}$-${\dot{V}}_{{\text{O}}_{2}}$ relationship with ${\dot{V}}_{{\text{O}}_{2}}$ as the dependent variable [89, 165, 169]. In this construct, higher ${\dot{V}}_{{\text{O}}_{2}}$ values (or steeper rates of change) for a given $\;{\dot{V}}_{\text{E}}$ would indicate a more “efficient” O_2_ uptake by the lungs. It should be emphasized, however, that exercise $\;{\dot{V}}_{\text{E}}$ is more closely related to ${\dot{V}}_{{\text{CO}}_{2}}$ than ${\dot{V}}_{{\text{O}}_{2}}$ [170] which makes the concept of ${\dot{V}}_{{\text{O}}_{2}}$ efficiency prone to misinterpretation (see Section 2.3.3). 

#### 2.3.2. Technical Considerations

**Figure 5:**
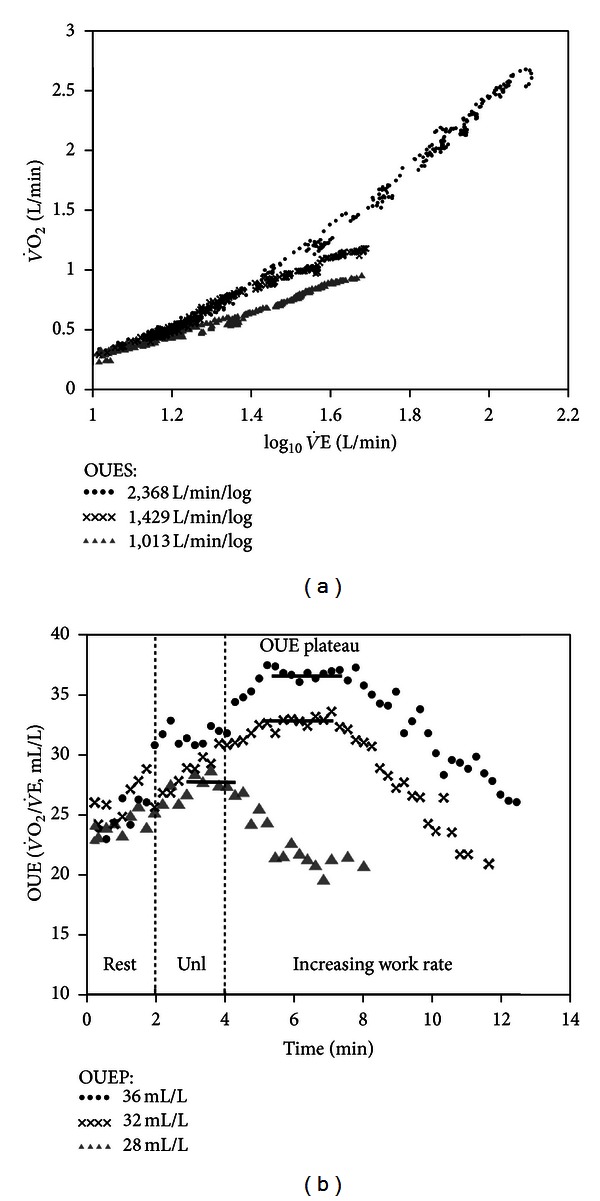
Relationship between oxygen uptake $\left({\dot{V}}_{{\text{O}}_{2}}\right)$ and minute ventilation $\left({\dot{V}}_{\text{E}}\right)$ during incremental exercise in a healthy subject (∙∙∙∙) and patients with mild (xxxx) and severe (▲▲▲▲) CHF. (a) The slope of ${\dot{V}}_{{\text{O}}_{2}}$ upon $\log_{10}{\dot{V}}_{\text{E}}$ is the oxygen uptake efficiency slope (OUES) which gives the rate of increase in ${\dot{V}}_{{\text{O}}_{2}}$ for a 10-fold rise in $\;{\dot{V}}_{\text{E}}$. (b) The highest ${\dot{V}}_{{\text{O}}_{2}}/{\dot{V}}_{\text{E}}$ ratio is the ${\dot{V}}_{{\text{O}}_{2}}$ efficiency slope (OUEP) which is the average of values just prior to the estimated lactate threshold. Unl is unloaded pedaling.

Baba and coworkers [165] proposed a logarithmic transformation of $\;{\dot{V}}_{\text{E}}$ over the entire exercise period to “linearize” this relationship, the so-called ${\dot{V}}_{{\text{O}}_{2}}$ efficiency slope (OUES) (Figure 5(a)). More recently, Sun et al. [89, 169] expressed the OUE as a ratio (${\dot{V}}_{{\text{O}}_{2}}/{\dot{V}}_{\text{E}}$ in mL/L) over time which, as expected, gives a mirror image of the ventilatory equivalent for O_2_. The authors proposed the term OUE plateau (OUEP) to the 90 s-average of the highest consecutive ${\dot{V}}_{{\text{O}}_{2}}/{\dot{V}}_{\text{E}}$ measurements; that is, the values just before the LT (Figure 5(b)). Although they reported that OUEP was more reproducible than OUES, this was not yet independently confirmed. It has been claimed that both relationships are independent of interobserver variability and effort [90, 164, 171–173]. However, Williamson et al. [173] recently found that there was a significant increase in OUES as exercise moved from low to moderate intensity with a peak value at an RER value of 1.0. Oscillatory breathing (see Section 3.3) has been found to interfere little with OUE estimations [89]. It should be recognized that both OUES and OUEP require separate computation though some commercially available CPET systems allow logarithmic transformations for OUES calculation.

#### 2.3.3. Interpretative Issues

It is well established that exercise hyperpnea is under stronger influence of *P*_a_CO_2_ and pH_a_ (rather than *P*_a_O_2_) [170]. As detailed later (Section 3.1), changes in CO_2_ set-point and ventilatory “efficiency” control the rate of CO_2_ clearance. This brings substantial uncertainty on the exact physiological meaning of a disturbed relationship between ${\dot{V}}_{\text{E}}$ and ${\dot{V}}_{{\text{O}}_{2}}$. Nevertheless, the literature pertaining to the clinical usefulness of OUES is rather vast in CHF [90, 164, 165, 167, 171, 172], and interest in this relationship has been spread to other populations (cystic fibrosis, and surgical candidates) [174, 175]. A number of studies have found that OUES is strongly correlated with ${\dot{V}}_{{\text{O}}_{2}}$ peak [90, 164, 165, 167, 171, 172, 176, 177] and may hold prognostic value in CHF [18, 89–94]. However, the prognostic advantage of OUES over $\Delta {\dot{V}}_{\text{E}}/\Delta {\dot{V}}_{\text{C}{\text{O}}_{2}}$ slope remains unclear [178, 179]. In the pediatric group, mixed results were reported and at least one study found that OUES determined at different WRs differed significantly within patients with cystic fibrosis and correlated only moderately with ${\dot{V}}_{{\text{O}}_{2}}$ peak and VT [180]. Interestingly, OUES showed to be more sensitive to the effects of training than $\Delta {\dot{V}}_{\text{E}}/\Delta {\dot{V}}_{\text{C}{\text{O}}_{2}}$ slope in patients with CHF [96], a finding correlated with enhanced cerebral and muscle hemodynamics in another study [95]. On a single investigation from the group which proposed OUEP, this relationship either on isolation or in combination with oscillatory breathing was prognostically superior to traditional key CPET parameters in CHF [89]. Predicting equations for OUES and OUEP have been recently published [169].

### 2.4. Postexercise ${\dot{V}}_{{\text{O}}_{2}}$

#### 2.4.1. Physiological Background

After ramp-incremental exercise,$\;{\dot{V}}_{{\text{O}}_{2}}$ does not decline immediately towards the resting level. The traditional view is that there would be a “debt payment” of energy deficit contracted at the start of effort (O_2_ deficit). Indeed, the time course of ${\dot{V}}_{{\text{O}}_{2}}$ recovery after a moderate, constant test has been found to track the rate of phosphocreatine resynthesis [181]. At early recovery, replenishment of local O_2_ sources in muscles (oxymyoglobin and dissolved O_2_) and reloading of haemoglobin are also needed [182]. At later stages, lactate metabolism (oxidation or gluconeogenesis) and increased cathecolamines and temperature also interfere with the dynamics of ${\dot{V}}_{{\text{O}}_{2}}$ decrease [183, 184].

#### 2.4.2. Technical Considerations

${\dot{V}}_{{\text{O}}_{2}}$ during recovery has been evaluated by (a) the ratio between total  ${\dot{V}}_{{\text{O}}_{2}}$ during exercise and recovery [185], (b) the time constant of ${\dot{V}}_{{\text{O}}_{2}}$ decay (i.e., time to reach 63% of the lowest value as obtained by fitting a decreasing monoexponential function) [182, 186, 187], (c) *t* ^1/2^ (time required for ${\dot{V}}_{{\text{O}}_{2}}$ to decrease to half of its peak value) [185, 188–190], and (d) ${\dot{V}}_{{\text{O}}_{2}}\;\;t$-slope (the response slope during the first minute of recovery by linear regression) [188, 189]. A further increase in ${\dot{V}}_{{\text{O}}_{2}}$ during recovery [191] (i.e., a ${\dot{V}}_{{\text{O}}_{2}}$ “overshoot”) has been found indicative of severe hemodynamic dysfunction as it reflects prolonged ${\dot{V}}_{{\text{O}}_{2}}$ kinetics [192, 193]. Importantly, the level of effort seems not critical for a valid analysis of post-exercise ${\dot{V}}_{{\text{O}}_{2}}$ dynamics [190].

#### 2.4.3. Interpretative Issues

**Figure 6:**
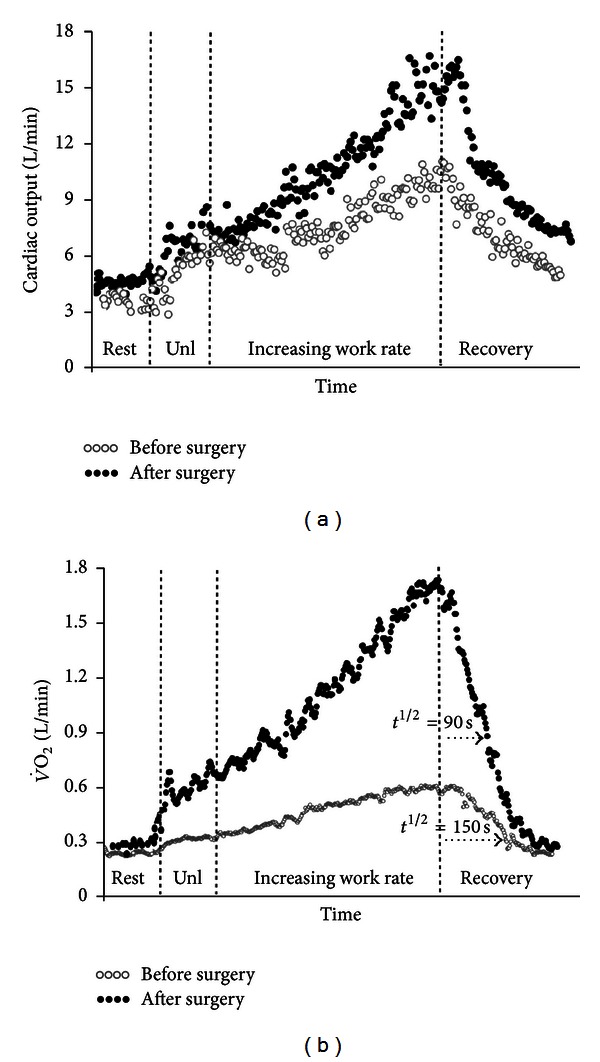
Incremental cycle ergometer exercise tests in the same patient of Figure 4 with chronic thromboembolic pulmonary hypertension. After pulmonary endarterectomy (*closed symbols*), haemodynamic improvement (*panel* (a)) led to a higher oxygen uptake $\left({\dot{V}}_{{\text{O}}_{2}}\right)$ at peak exercise and a faster (lower half-time (*t* ^1/2^) post-exercise decrease in ${\dot{V}}_{{\text{O}}_{2}}$ (*panel* (b)). Cardiac output was noninvasively estimated by impedance cardiography and the tests were time-aligned by total exercise duration. Unl is unloaded pedaling.

Delayed ${\dot{V}}_{{\text{O}}_{2}}$ recovery has been related to functional impairment in CHF [188, 189, 192, 194], myocardial ischemia [195], COPD [196], and functional impairment in several conditions, including cystic fibrosis [197], diabetes [198], deconditioning [199], and obstructive sleep apnea [139]. Impairment in cardiovascular responses to exercise as indicated by a delayed recovery of cardiac output was closely associated with slower off-exercise ${\dot{V}}_{{\text{O}}_{2}}$ kinetics in CHF [200]. Improvements in O_2_ delivery might be expected to speed the rate of O_2_ recovery in cardiovascular diseases (Figure 6) [201].

## 3. Ventilatory Responses

### 3.1. Excess Exercise Ventilation

#### 3.1.1. Physiological Background

Adequate increases in alveolar ventilation (${\dot{V}}_{\text{A}}$) are paramount to wash out metabolically produced CO_2_. Exercise ${\dot{V}}_{\text{E}}$ for a given ${\dot{V}}_{\text{C}{\text{O}}_{2}}$ is inversely related to the prevailing level at which *P*_a_CO_2_ is regulated (the CO_2_ “set-point”) and the dead space (*V*_D_)/tidal volume (*V*_T_) ratio; that is,

(2) $$\begin{array}{c} \frac{{\dot{V}}_{\text{E}}}{{\dot{V}}_{\text{C}{\text{O}}_{2}}}=\frac{1}{{P_{\text{a}}\text{CO}}_{2}\left(1-\left(V_{\text{D}}/V_{\text{T}}\right)\right)}. \end{array}$$

Consequently, the largest $\;{\dot{V}}_{\text{E}}/{\dot{V}}_{{\text{CO}}_{2}}$values will be found in those who chronically hyperventilate (low CO_2_ “set-point”) and have the large *V*_D_ coupled with a low *V*_T_ [202–206]. In the clinical literature, an increased slope of the $\;{\dot{V}}_{\text{E}}$-${\dot{V}}_{\text{C}{\text{O}}_{2}}$ relationship has been termed ventilatory “inefficiency” though it could be argued that there is no “inefficiency” when increased $\;{\dot{V}}_{\text{E}}$ results from alveolar hyperventilation. “Excess exercise ventilation” seems therefore a more appropriated description of a greater-than-expected ventilatory response to metabolic demand [205]. 

#### 3.1.2. Technical Considerations

**Figure 7:**
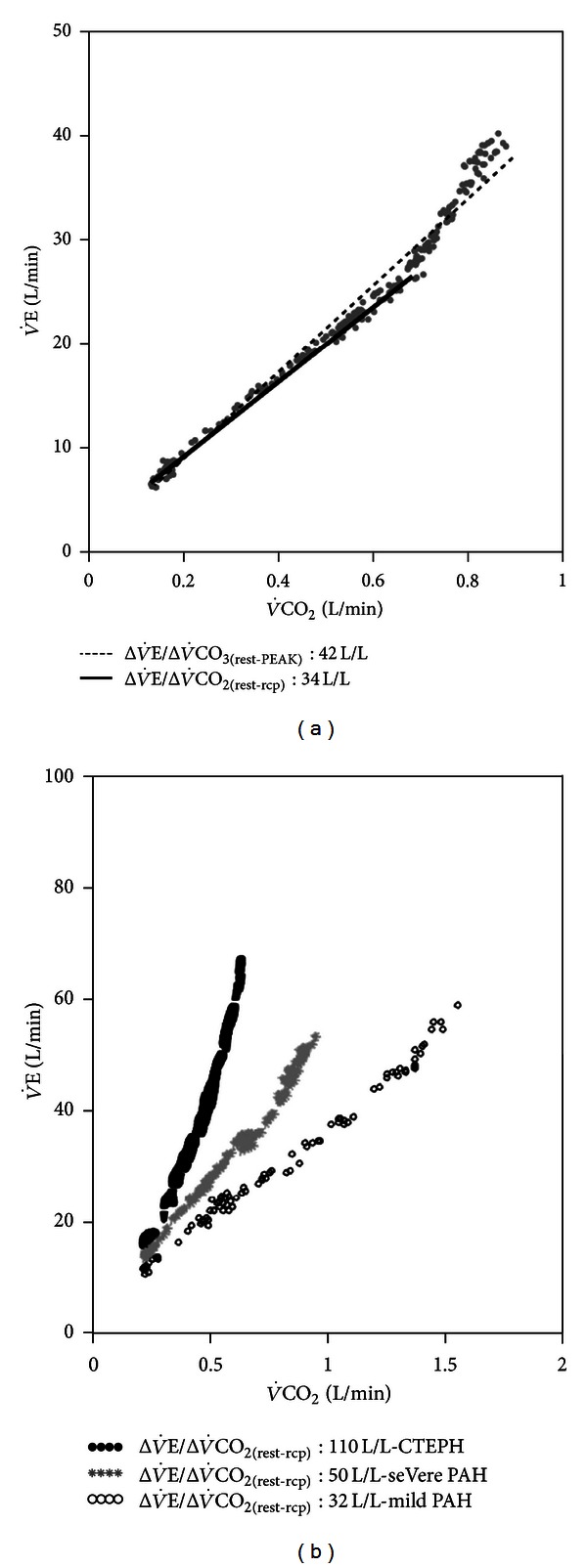
(a) Minute ventilation $({\dot{V}}_{\text{E}})/$carbon dioxide output (${\dot{V}}_{{\text{CO}}_{2}}$) relationship from the beginning of exercise to the respiratory compensation point (solid line) or up to peak exercise (dashed line) in a patient with CHF. Note that $\Delta {\dot{V}}_{\text{E}}/\Delta {\dot{V}}_{{\text{CO}}_{2(\text{rest-PEAK})}}$ is steeper than $\Delta {\dot{V}}_{\text{E}}/\Delta {\dot{V}}_{{\text{CO}}_{2(\text{rest-RCP})}}$ because it adds a component of hyperventilation to lactic acidosis and/or other stimuli after the respiratory compensation point. (b) $\Delta {\dot{V}}_{\text{E}}/\Delta {\dot{V}}_{{\text{CO}}_{2}}$ as a function of disease severity in pulmonary arterial hypertension (PAH). Higher values, however, are usually found in chronic thromboembolic pulmonary hypertension (CTEPH) due to pronounced increases in tidal volume ratio.

**Figure 8:**
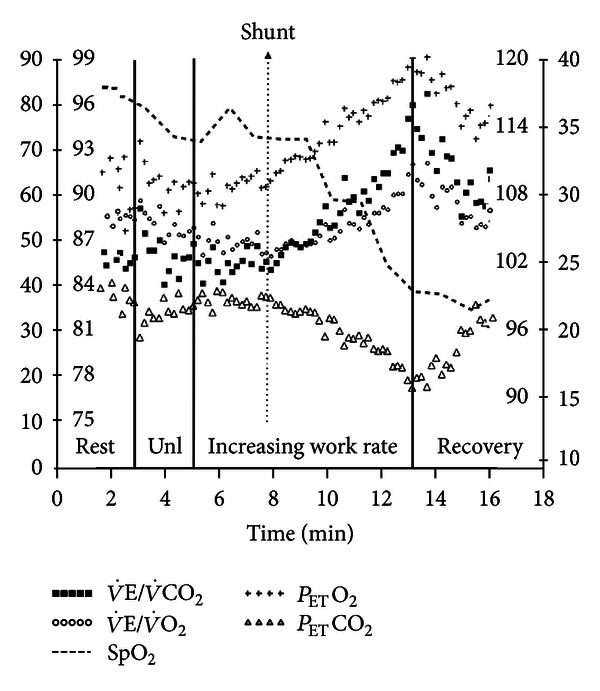
Exercise-induced right-to-left shunt as suggested by sudden decrease in oxyhemoglobin saturation by pulse oximetry (SpO_2_) and abrupt increases in the ventilatory equivalents for CO_2_ and O_2_ (${\dot{V}}_{\text{E}}/{\dot{V}}_{{\text{CO}}_{2}}$ and $\;{\dot{V}}_{\text{E}}/{\dot{V}}_{{\text{O}}_{2}}$) associated with a sustained decrease in the end-tidal partial pressure for CO_2_ (*P*_ET_CO_2_) with a concomitant increase in *P*_ET_O_2_ in a patient with pulmonary arterial hypertension. Shunting of systemic venous blood in the arterial circulation stimulated the peripheral chemoreceptors thereby leading to this pattern of ventilatory and gas exchange responses. Unl is unloaded pedaling.

There are a number of alternatives to express the $\;{\dot{V}}_{\text{E}}$-${\dot{V}}_{\text{C}{\text{O}}_{2}}$ relationship during progressive exercise: (1) as a ratio ($\;{\dot{V}}_{\text{E}}/{\dot{V}}_{\text{C}{\text{O}}_{2}}$) at peak exercise, at the VT (Figure 1(b)), and as the lowest (nadir) value and (2) as a slope of ${\dot{V}}_{\text{E}}$ versus ${\dot{V}}_{\text{C}{\text{O}}_{2}}$ from the beginning of exercise to the RCP ($\Delta {\dot{V}}_{\text{E}}/\Delta {\dot{V}}_{\text{C}{\text{O}}_{2\text{(rest-RCP)}}}$) (Figure 2(c)) or, alternatively, up to peak exercise ($\Delta {\dot{V}}_{\text{E}}/\Delta {\dot{V}}_{\text{C}{\text{O}}_{2\text{(rest-PEAK)}}}$) (Figure 7) [25, 26, 207]. Sun et al. reported that the $\;{\dot{V}}_{\text{E}}/{\dot{V}}_{\text{C}{\text{O}}_{2\text{(nadir)}}}$ had the least variability with the advantage that choosing the lowest value does not require VT identification [26]. However,$\;{\dot{V}}_{\text{E}}/{\dot{V}}_{\text{C}{\text{O}}_{2}}$ might not decline at all during early exercise in some patients with severe cardiopulmonary disease (Figure 8) which might preclude LT identification. *P*_a_CO_2_ is relatively constant up to the RCP, and, as described (2), a steeper-than-normal $\Delta {\dot{V}}_{\text{E}}/\Delta {\dot{V}}_{\text{C}{\text{O}}_{2\text{(rest-RCP)}}}$ can be explained by a higher *V*_D_/*V*_T_ and/or a low CO_2_ set point. $\Delta {\dot{V}}_{\text{E}}/\Delta {\dot{V}}_{\text{C}{\text{O}}_{2\text{(rest-PEAK)}}}$ is expected to be even steeper than $\Delta {\dot{V}}_{\text{E}}/\Delta {\dot{V}}_{\text{C}{\text{O}}_{2\text{(rest-RCP)}}}$ (Figure 7(a)) because the former adds a component of hyperventilation to lactic acidosis and/or to other sources of $\;{\dot{V}}_{\text{E}}$ stimuli at near maximum exercise [26, 207]. It should be emphasized, however, that there are interpretational pitfalls of using $\Delta {\dot{V}}_{\text{E}}/\Delta {\dot{V}}_{\text{C}{\text{O}}_{2\text{(rest-PEAK)}}}$ as a single linear characterization of a relationship which is characteristically curvilinear (Figure 7).$\;\;{\dot{V}}_{\text{E}}/{\dot{V}}_{\text{C}{\text{O}}_{2\text{nadir}}}$ is equal to $\Delta {\dot{V}}_{\text{E}}/\Delta {\dot{V}}_{\text{C}{\text{O}}_{2\text{(rest-RCP)}}}$ when the slope has an *y*-intercept of zero. However, $\Delta {\dot{V}}_{\text{E}}/\Delta {\dot{V}}_{\text{C}{\text{O}}_{2\text{(rest-RCP)}}}$ has a positive *y*-intercept in normal subjects [208] which explains why $\;{\dot{V}}_{\text{E}}/{\dot{V}}_{\text{C}{\text{O}}_{2\text{VT}}}$ is usually greater than the slope.$\;\;{\dot{V}}_{\text{E}}/{\dot{V}}_{\text{C}{\text{O}}_{2\text{VT}}}$ will also exceed the slope if the VT is a low value (i.e., in less fit subjects) [10]. On the other hand, a very steep $\Delta {\dot{V}}_{\text{E}}/\Delta {\dot{V}}_{\text{C}{\text{O}}_{2\text{(rest-RCP)}}}$ would produce a negative *y*-intercept thereby making it greater than $\;{\dot{V}}_{\text{E}}/{\dot{V}}_{\text{C}{\text{O}}_{2\text{VT}}}$ [205]. 

#### 3.1.3. Interpretative Issues

$\Delta {\dot{V}}_{\text{E}}/\Delta {\dot{V}}_{{\text{CO}}_{2(\text{rest-RCP})}}$ in healthy young males is approximately 30 [25, 26]; however, it increases with age probably as a result of larger *V*_D_/*V*_T_ in older subjects [10, 11]. Females have lower *V*_T_ for a given $\;{\dot{V}}_{\text{E}}$ than males independent of senescence which might explain their higher $\Delta {\dot{V}}_{\text{E}}/\Delta {\dot{V}}_{{\text{CO}}_{2(\text{rest-RCP})}}$ across all age ranges (Figure 3(c)) [10, 11]. There is plenty of evidence that $\Delta {\dot{V}}_{\text{E}}/\Delta {\dot{V}}_{{\text{CO}}_{2(\text{rest-RCP})}}$ is clinically useful as a prognostic marker in CHF [52, 108, 109, 163, 209–212] and, more recently, in PAH [97, 98, 213] with more discriminatory information than ${\dot{V}}_{{\text{O}}_{2}}$ peak. The prognostic value in CHF persisted in patients on *β*-blockers [99, 100]. Interestingly, $\Delta {\dot{V}}_{\text{E}}/\Delta {\dot{V}}_{\text{C}{\text{O}}_{2\text{(rest-PEAK)}}}$ has been found better than $\Delta {\dot{V}}_{\text{E}}/\Delta {\dot{V}}_{{\text{CO}}_{2(\text{rest-RCP})}}$ to predict 1-year cardiac mortality and hospitalization in these patients [207]. As expected, composite scores adding $\Delta {\dot{V}}_{\text{E}}/\Delta {\dot{V}}_{\text{C}{\text{O}}_{2}}$ to other cardiopulmonary variables improved even further their prognostic value [211]. A single study found that coexistence of COPD tends to “normalize” $\Delta {\dot{V}}_{\text{E}}/\Delta {\dot{V}}_{\text{C}{\text{O}}_{2}}$ in CHF patients which casts doubt on its prognostic usefulness in this specific subpopulation [214].

In patients with PAH, $\Delta {\dot{V}}_{\text{E}}/\Delta {\dot{V}}_{\text{C}{\text{O}}_{2}}$ and $\;{\dot{V}}_{\text{E}}/{\dot{V}}_{\text{C}{\text{O}}_{2}}$ (at rest, VT, and peak) are higher compared to CHF [215]. $\;{\dot{V}}_{\text{E}}/{\dot{V}}_{{\text{CO}}_{2\text{VT}}}$ > 37 *plus P*_ET_CO_2VT_ < 30 mmHg increased the probability of pulmonary vascular disease [111]. In those with idiopathic PAH, higher $\Delta {\dot{V}}_{\text{E}}/\Delta {\dot{V}}_{\text{C}{\text{O}}_{2}}$ and $\;{\dot{V}}_{\text{E}}/{\dot{V}}_{\text{C}{\text{O}}_{2}}$ (VT and nadir) were related to clinical [53] and hemodynamic impairment [104]. Importantly, these indexes improved with specific treatment [104, 105] and after pulmonary endarterectomy [106]. Although to date there is a lack of evidence that indices of excess exercise ventilation in PAH hold the same prognostic importance as in CHF, Deboeck et al. recently described that $\;{\dot{V}}_{\text{E}}/{\dot{V}}_{{\text{CO}}_{2\text{VT}}}$ (and the 6-min walking distance) were independent predictors of death [98]. Oudiz et al., however, found that $\;{\dot{V}}_{\text{E}}/{\dot{V}}_{\text{C}{\text{O}}_{2}}$ was valuable to prognosis assessment only when exercise-induced right-to-left shunt (Figure 8) was absent [119]. Although $\;{\dot{V}}_{\text{E}}/{\dot{V}}_{\text{C}{\text{O}}_{2}}$ is particularly disturbed in chronic thromboembolic pulmonary hypertension (CTEPH) (Figure 7(b)), thrombotic vessels occlusion increases *V*_D_/*V*_T_ and excess exercise ventilation to levels which may not be proportionately related to hemodynamic impairment [216].

In patients with other chronic respiratory diseases, $\Delta {\dot{V}}_{\text{E}}/\Delta {\dot{V}}_{{\text{CO}}_{2(\text{rest-RCP})}}$ > 34 increased the risk of post-operative complications after lung resection surgery with better prediction power than ${\dot{V}}_{{\text{O}}_{2}}$ peak and predicted post-operative ${\dot{V}}_{{\text{O}}_{2}}$ peak [110]. It could also be empirically expected that a low $\;{\dot{V}}_{\text{E}}/{\dot{V}}_{{\text{CO}}_{2\text{VT}}}$ would be rarely associated with increased *V*_D_/*V*_T_ whereas the opposite would be likely at very high $\;{\dot{V}}_{\text{E}}/{\dot{V}}_{{\text{CO}}_{2\text{VT}}}$. In fact, Roman and coworkers recently described that when $\;{\dot{V}}_{\text{E}}/{\dot{V}}_{{\text{CO}}_{2\text{VT}}}$ was ≤28 and within 29–32, 96% and 83% of subjects had normal *V*_D_/*V*_T_. On the other hand, *V*_D_/*V*_T_ was abnormal in 87% of the cases when $\;{\dot{V}}_{\text{E}}/{\dot{V}}_{{\text{CO}}_{2\text{VT}}}$ was ≥39. Unfortunately, intermediate values were not useful to discriminate the underlying mechanisms. Interestingly, 95% of the patients with an obstructive ventilatory defect (FEV_1_/FVC < 0.7) and $\;{\dot{V}}_{\text{E}}/{\dot{V}}_{{\text{CO}}_{2\text{VT}}}$ ≥ 39 had increased *V*_D_/*V*_T_ [217].

### 3.2. End-Tidal Partial Pressure for CO_2_

#### 3.2.1. Physiological Background

Expired CO_2_ concentration increases as air from the serial (“anatomic”) *V*_D_ is progressively enriched with CO_2_ from the gas exchanging areas. Consequently, the largest partial pressures for CO_2_ are found at the end of tidal expiration (*P*_ET_CO_2_). However, *P*_ET_CO_2_ is influenced not only by the metabolic rate (i.e., the rate of increase in mixed venous *P*_CO_2__) but also by the deepness of the previous inspiration (i.e., VT) and the duration of the exhalation. *P*_ET_CO_2_ reflects poorly *P*_a_CO_2_, (ideal alveolar) as there are significant regional variations in alveolar *P*_CO_2__ (*P*_A_CO_2_) and ${\dot{V}}_{\text{A}}$-to-perfusion ratios—even in normal subjects [2, 16]. It should also be recognized that *P*_ET_CO_2_ becomes systematically greater than *P*_a_CO_2_ during incremental exercise as the first is the peak of the intrabreath oscillation of *P*_A_CO_2_ and *P*_a_CO_2_ measured in peripheral arterial blood is an average of the oscillation over several breaths [2, 16].

#### 3.2.2. Technical Considerations

**Figure 9:**
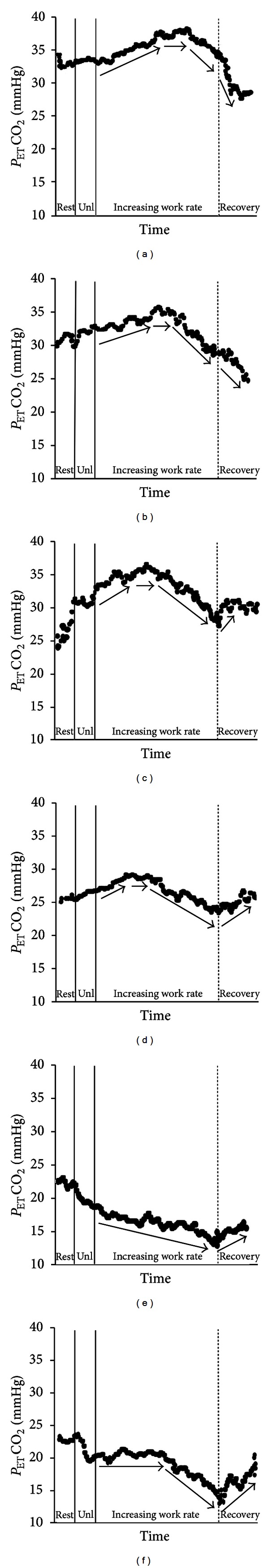
Time course of end-tidal partial pressure for carbon dioxide (*P*_ET_CO_2_) during incremental exercise and early recovery in a healthy control (*panel *(a)) and five patients with pulmonary arterial hypertension of progressing severity (*panels *(b)* to *(f)). Note that *P*_ET_CO_2_ becomes lower and even fails to increase as disease progresses. Moreover, it frequently increases (instead of diminishing) during recovery. *Panel *(f), in particular, depicts a severely impaired patient showing abrupt and sustained decrease in *P*_ET_CO_2_ concomitant with the opening of a *forame ovale *(Figure 8). Unl is unloaded pedaling.

*P*_ET_CO_2_ increases from rest to LT (which is proportional to decrease in$\;{\dot{V}}_{\text{E}}/{\dot{V}}_{{\text{CO}}_{2}}$) in this time range, followed by a stable phase during the isocapnic buffering period, and then a fall after the RCP (Figures 1(b) and 9(a)). As mentioned, *P*_a_CO_2_ underestimation by *P*_ET_CO_2_ is roughly proportional to *V*_D_/*V*_T_; consequently, computing *V*_D_/*V*_T_ using *P*_ET_CO_2_ instead of *P*_a_CO_2_ overestimates *V*_D_/*V*_T_ in normal subjects and underestimates it in patients [218]. 

#### 3.2.3. Interpretative Issues

*P*_ET_CO_2_ differs from *P*_a_CO_2_ as a result of ventilation-to-perfusion inhomogeneities, right-to-left shunt, and changes in breathing pattern [2, 16]. However, arterial blood gases are not routinely measured during clinical CPET. Consequently, interpretation of a reduced *P*_ET_CO_2_ is complex in the absence of *P*_a_CO_2_ measurements as it might be related to abnormal gas exchange, alveolar hyperventilation, or a tachypneic and shallow pattern of breathing. Regardless of the exact mechanism, abnormally low values at the LT have been found useful for the diagnosis of pulmonary vascular diseases in patients with unexplained dyspnea [111]. There is now established evidence that *P*_ET_CO_2_ at rest [112–114], LT [115], and peak exercise [116] are valuable for prognosis estimation and disease severity assessment in CHF [219, 220]. Low *P*_ET_CO_2_ values have also been found in PAH (see also later) [97, 111, 117, 118]. Decreased *P*_ET_CO_2_ at rest and during exercise seems to track the blunted cardiac output response to exercise in cardiovascular disease [219, 221]. Accordingly, exercise training after acute myocardial infarction increases both *P*_ET_CO_2_ and cardiac output [120]. In addition to reduced cardiac output, an augmented ventilatory drive may also account for a reduction in *P*_ET_CO_2_ whereas altered breathing pattern seems to have a minor role in CHF [204].

*P*_ET_CO_2_ is typically lower in PAH than CHF [111, 219]. In fact, Yasunobu and co-workers suggested that observation of an unusually low *P*_ET_CO_2_ at the LT (<30 mmHg or, in particular, <20 mmHg) in a patient with exertional dyspnea of unknown cause without evidence of acute hyperventilation (ie, normal R) should prompt the hypothesis of pulmonary vasculopathy [111]. *P*_ET_CO_2_ response profile is also informative as failure to increase below the LT or progressive decreases from the start of exercise are associated with worsening clinical and hemodynamic impairment (Figures 9(b) to 9(e)) [111] and are rarely found in CHF [112–116]. Based on (2), it might be expected that if *P*_ET_CO_2_ changed parallel to *P*_A_CO_2_, a hyperbolic relationship between $\;{\dot{V}}_{\text{E}}/{\dot{V}}_{{\text{CO}}_{2}}$ and *P*_ET_CO_2_ at the LT would result. As this was observed by Yasunobu et al. [111] and confirmed by others [104, 216], it seems that alveolar hyperventilation is an important contributing mechanism to the excess exercise ventilation in PAH. Moreover, sharp decreases in *P*_ET_CO_2_ may indicate exercise-induced intracardiac shunt, a finding with ominous consequences (Figures (8) and 9(f)) [119]. Additionally, an abnormal increase in *P*_ET_CO_2_ during early recovery has been described in PAH (Figure 9(c)), even in mildly-impaired patients [111].

### 3.3. Exertional Oscillatory Ventilation (EOV)

#### 3.3.1. Physiological Background

An abnormal pattern of ventilation consisting of cyclic hyperpnea and hypopnea without interposed apneas can be detected by CPET in some patients with advanced CHF. The EOV might occur throughout the test, but the oscillations frequently dampen as exercise progresses [121, 222–224]. The pathophysiological mechanisms are multifactorial including low cardiac output leading to a prolonged time of pulmonary venous blood to reach the central or peripheral chemoreceptors, low lung volume, pulmonary congestion, augmented chemoreceptor sensitivity, and the narrow difference between the eupneic *P*_a_CO_2_ and the apneic (or hypoventilatory) threshold [27, 122, 123, 225–235].

#### 3.3.2. Technical Considerations

**Figure 10:**
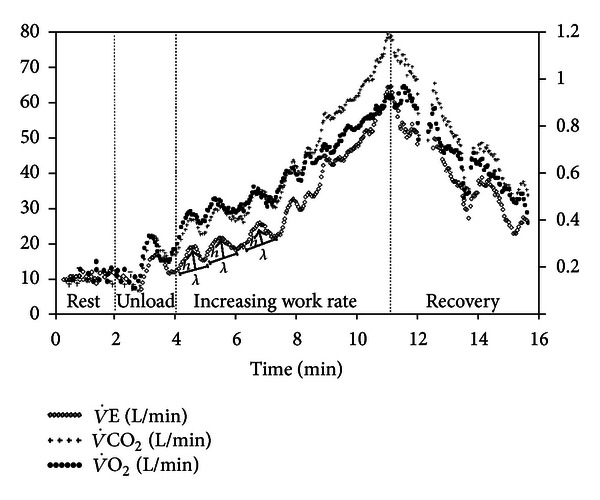
Exertional oscillatory ventilation (EOV) during incremental CPET in a 56-yr-old male with severe CHF. EOV was defined by regular (standard deviation of three consecutive cycle lenghts (*λ*) within 20% of their average) and ample (minimal *h* of 5 L/min) cycles of ventilatory $\left({\dot{V}}_{\text{E}}\right)$ oscillations [27]. A similar oscillatory pattern is also seen in oxygen uptake $\left({\dot{V}}_{{\text{O}}_{2}}\right)$ and carbon dioxide output $\left({\dot{V}}_{{\text{CO}}_{2}}\right)$.

Different criteria for EOV might help explaining why its prevalence has been found to vary from 12% to 50% in CHF [123, 124, 236–238]. A widely used definition is as follows (Figure 10): (1) three or more regular oscillations (i.e., clearly discernible from inherent data noise); (2) standard deviation of three consecutive cycle lengths (time between 2 consecutive nadirs) within 20% of the average; (3) minimal average amplitude of ${\dot{V}}_{\text{E}}$ oscillation of 5 L/min (peak value *minus* the average of two in-between consecutive nadirs) [27]. Alternative definitions require: (i) criteria for persistence of the EOV pattern (three or more consecutive cyclic oscillations) for at least 60% of exercise at an amplitude ≥ 15% of the average resting value [122, 239–241] or (ii) 3 or more consecutive cyclic fluctuations with amplitude exceeding 30% of mean $\;{\dot{V}}_{\text{E}}$ and oscillatory cycle within 40 to 140 s in 3 or more gas exchange/ventilatory variables [124].

#### 3.3.3. Clinical Usefulness

There is now well-established evidence that EOV holds important negative prognostic implications in patients with CHF [27, 124, 222, 236, 239], being related to worsening clinical status [121, 122, 124], severe hemodynamic dysfunction [123], and reduced functional capacity [125, 126]. Unfortunately, EOV may preclude an adequate identification of the LT by either the *V-slope* or the ventilatory equivalent methods [242]. EOV is highly reproducible regardless of the CHF aetiology [121]. Interestingly, several interventions including inotropics [237], exercise and inspiratory muscle training [243–245], and transplantation [237] lessened of even abolished EOV. Future larger trials should establish whether EOV might add independent information to commonly used outcomes for interventional studies in CHF.

## 4. Cardiovascular Responses

### 4.1. Δ Heart Rate (HR)/Δ Oxygen Uptake $\left({\dot{V}}_{{\text{O}}_{2}}\right)$

#### 4.1.1. Physiological Background

**Figure 11:**
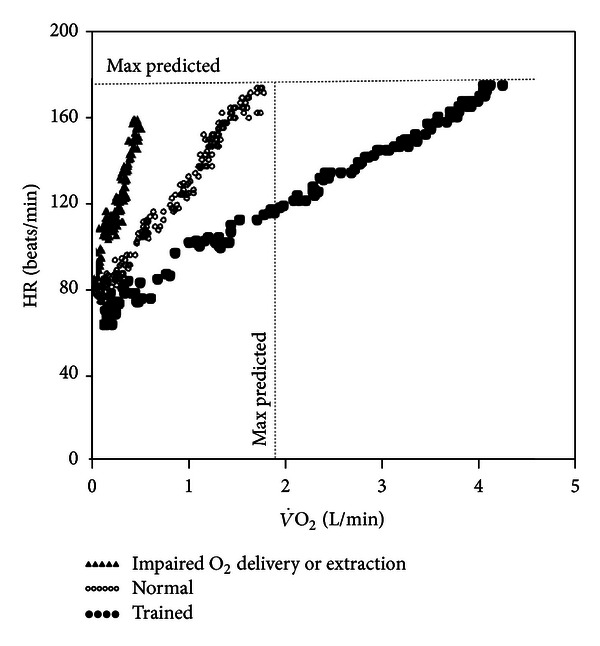
Heart rate (HR) response as a function of O_2_ uptake $\left({\dot{V}}_{{\text{O}}_{2}}\right)$ in 3 males of same age: a patient with abnormal O_2_ delivery and/or extraction (severe pulmonary arterial hypertension, $\Delta \text{HR}/\Delta {\dot{V}}_{{\text{O}}_{2}}$ = 158  beats/L), a normal sedentary subject ($\Delta \text{HR}/\Delta {\dot{V}}_{{\text{O}}_{2}}$ = 65  beats/L), and a triathlete ($\Delta \text{HR}/\Delta {\dot{V}}_{{\text{O}}_{2}}$ = 26  beats/L).

Increases in HR with progressive exercise are initially mediated by parasympathetic tonus withdrawal and, subsequently, by increased sympathetic activity [246]. There is an effectively linear increase in HR as a function of ${\dot{V}}_{{\text{O}}_{2}}$ during ramp-incremental exercise [3, 24, 25] though departs from linearity might occur at higher exercise intensities (Figure 2(b)) [247]. According to the *Fick* principle, reduced stroke volume (SV) and/or diminished C(a–v)O_2_ would lead to a steeper $\Delta \text{HR}/\Delta {\dot{V}}_{{\text{O}}_{2}}$ slope. Consequently, cardiac dysfunction, decreased arterial O_2_ content (anemia and hypoxemia), and impaired muscle aerobic capacity (e.g., deconditioning, mitochondrial dysfunction) can potentially increase $\Delta \text{HR}/\Delta {\dot{V}}_{{\text{O}}_{2}}$. On the other hand, training has a flattening effect on $\Delta \text{HR}/\Delta {\dot{V}}_{{\text{O}}_{2}}$ (Figure 11).

#### 4.1.2. Technical Considerations

Although ${\dot{V}}_{{\text{O}}_{2}}$ is the appropriate dependent variable, this relationship has been traditionally described with HR on the *y*-axis [3, 24, 25]. Linearity of the HR response throughout the test duration should be firstly established. In event of late departures from linearity, the slope should be calculated only over the initial linear phase response (Figure 2(b)). As detailed later, pronounced changes in linearity may hold important clinical implications.

#### 4.1.3. Clinical Usefulness

**Figure 12:**
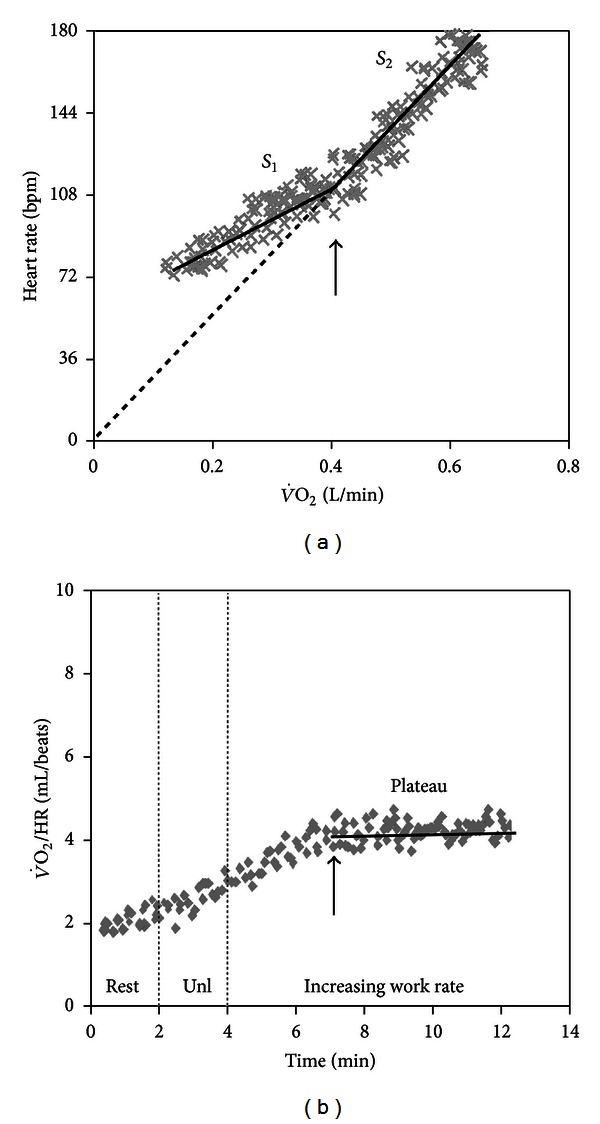
Change in Δ heart rate (HR)/Δ oxygen uptake $\left({\dot{V}}_{{\text{O}}_{2}}\right)$ (*arrow*) slope (*arrow*) during incremental CPET in a patient with severe cardiovascular limitation to exercise (*panel* (a)). Note that this led to a plateau in O_2_ pulse (${\dot{V}}_{{\text{O}}_{2}}/\text{HR}$ ratio) as the *y*-intercept becomes zero; that is, the relationship passes through its origin (*panel* (b)). Unl is unloaded pedaling.

**Figure 13:**
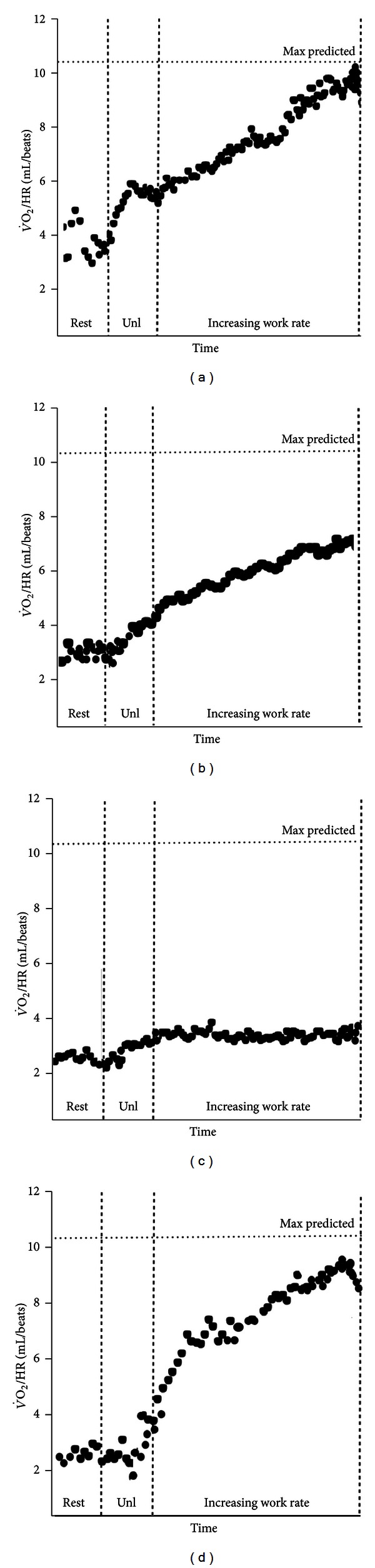
O_2_ pulse $\left({\dot{V}}_{{\text{O}}_{2}}/\text{HR}\right)$ as a function of time during incremental exercise. (a) Curvilinear increase up to a normal predicted value in a healthy subject; (b) abnormally low peak values due to ventilatory limitation and early exercise cessation in a patient wirh COPD; (c) failure to increase and early plateau in a patient with end-stage pulmonary arterial hypertension; (d) decrease at near maximum exercise in a patients with concomitant electrocardiographic abnormalities indicative of coronary artery disease. Unl is unloaded pedaling.

$\Delta \text{HR}/\Delta {\dot{V}}_{{\text{O}}_{2}}$ increases with age being consistently higher in females than males (Figure 3(b)) [10]. As expected, cardiovascular and muscular diseases which are known to impair O_2_ delivery and/or utilization have been found to increase both the slope and the intercept of the $\Delta \text{HR}/\Delta {\dot{V}}_{{\text{O}}_{2}}$ relationship [127–130]. Some specific conditions, however, may prevent HR to increase even in the presence of disease: (a) patients under *β*-blocker therapy [248], (b) ischemic involvement of the sinusal node artery [249], and (c) advanced CHF [250]. The so-called O_2_ pulse (${\dot{V}}_{{\text{O}}_{2}}/\text{HR}$ ratio) is a commonly used derivation of $\Delta \text{HR}/\Delta {\dot{V}}_{{\text{O}}_{2}}$. As the primary ${\dot{V}}_{{\text{O}}_{2}}$-HR relationship has a negative *y*-intercept, O_2_ pulse increases hyperbolically [16] towards an asymptotic value at end-exercise (Figure 13(a)) which might reflect the SV response [131]. However, all pathologic conditions known to increase $\Delta \text{HR}/\Delta {\dot{V}}_{{\text{O}}_{2}}$ (including desaturation, anemia, and impaired O_2_ extraction) will also diminish peak O_2_ pulse. Moreover, early exercise termination due to symptom limitation (including breathlessness in patients with COPD) (Figure 13(b)) and/or submaximal effort would decrease peak O_2_ pulse in the absence of cardiovascular limitation. In these cases, however, a normal $\Delta \text{HR}/\Delta {\dot{V}}_{{\text{O}}_{2}}$ is reassuring. A more clinically useful pattern of response relates to abrupt increases in $\Delta \text{HR}/\Delta {\dot{V}}_{{\text{O}}_{2}}$ slope to an extent that the relationship goes through its origin or becomes with a negative *y*-intercept; that is, O_2_ pulse turns flat (Figure 12) or even decreases (Figure 13(d)). This suggests that the HR response became the sole mechanism for cardiac output increase due to a severely impaired SV response. In practical grounds, there is limited evidence that as myocardial perfusion is reduced in patients with coronary artery disease, there is reversible left ventricle dysfunction thereby steepening $\Delta \text{HR}/\Delta {\dot{V}}_{{\text{O}}_{2}}$ (Figure 12(a)) and flattening (Figure 12(b)) (or even decreasing) (Figure 13(d)) O_2_ pulse [88, 132, 133].

### 4.2. Heart Rate Recovery (HRR)

#### 4.2.1. Physiological Background

At the start of exercise, HR increases as a result of early parasympathetic withdrawal and subsequent sympathetic activation [246]. After effort cessation, vagal reactivation (with opposition of the sympathetic drive) is primarily responsible for the return to baseline conditions [251], especially during the first 30 seconds of recovery [252]. Consequently, autonomic imbalance (increased sympathetic stimuli and/or impaired parasympathetic activity) might slow post-exercise HR decay. 

#### 4.2.2. Technical Considerations

HRR is the difference between peak HR and HR at selected time points after exercise (e.g., 30 sec and every minute thereafter). HRR analysis may be performed independent of the mode of exercise (treadmill [134, 135, 140, 152, 253], cycle ergometer [28, 254–256], or field tests [257]), and a cool-down period at the end of maximal effort seems not to interfere with its prognostic value [28, 134, 150].

#### 4.2.3. Interpretative Issues

**Figure 14:**
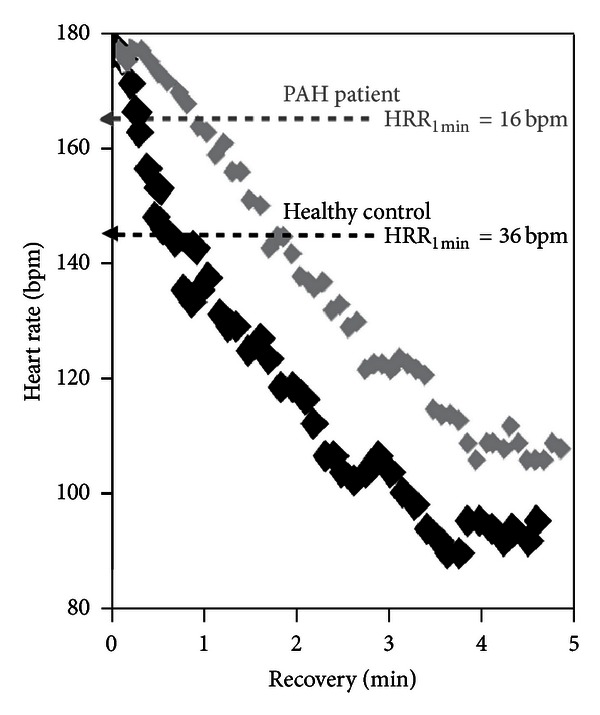
Heart rate (HR) response after incremental exercise in a healthy control and a patient with pulmonary arterial hypertension (PAH) of same age and gender (both females aged 31). Note the delayed HR recovery (HRR) up to the 5th minute after-exercise in the patient compared to the control. HRR_1 min_ ≤ 18 bpm after cycle ergometer exercise test has recently been found an independent predictor of mortality in these patients [28].

HRR has been found a simple and inexpensive prognostic marker in healthy populations [134], CHF [135], CAD [151, 258], PAH [28] (Figure 14), diabetes mellitus [136], and COPD [137]. Abnormal HRR has also been demonstrated in other systemic disorders such as metabolic syndrome [138], obstructive sleep apnea [139], sarcoidosis [140], rheumatological diseases [141, 142], polycystic ovary syndrome [143], polycystic kidney disease [144], and HIV infection [145]. Of note, it has been useful for risk stratification in CHF patients with mildly reduced peak ${\dot{V}}_{{\text{O}}_{2}}$ [259]. HRR seems to be responsive to exercise training in some disorders [146–149], probably due to effects of exercise on autonomic regulation [260, 261]. Interestingly, these modifications were related to increased survival after rehabilitation in patients with previous myocardial infarction [262, 263].

## 5. Conclusions

Interpretation of incremental CPET is best performed by a judicious analysis of all available physiological information provided by the procedure (and by previous testing) taking into consideration the underlying clinical question(s). Although a considerable lack of information on the individual diagnostic and prognostic value of the dynamic sub-maximal relationships still persists, the bulk of evidence is reassuring in relation to their practical usefulness. Large-scale, multicentric studies, however, are urgently needed to validate the suggested cutoffs of abnormality (Table 1) in different clinical scenarios and disease populations. 

## Figures and Tables

**Table 1:** Clinical usefulness and suggested cutoffs of selected dynamic responses to rapidly incremental CPET.

Variable	Clinical usefulness	Cutoffs/patterns of abnormality
Metabolic		

Estimated lactate threshold (LT)	(i) Prognosis in CHF [52]	(i) ${\dot{V}}_{{\text{O}}_{2}}$ LT < 40% predicted ${\dot{V}}_{{\text{O}}_{2}}$ peak [2]
	(ii) Marker of disease severity in PAH [53]	(ii) Influenced by age, gender, and fitness [4, 7, 42, 76]
	(iii) Risk predictor of postoperatory complications in the elderly [50, 51]	
	(iv) Guide exercise training intensity [72, 73]	
	(v) Responsive to rehabilitation in less impaired patients with chronic cardiopulmonary diseases [54, 70]	

$\Delta {\dot{V}}_{{\text{O}}_{2}}$/Δ work rate (mL/min/W)	(i) Indicative of impaired O_2_ delivery and/or utilization [77–81]	(i) <lower limit of normality (<8.5 mL/min/W) [4, 8]
	(ii) Adjunct for the diagnosis of myocardial ischemia [82–88]	(ii) Decrease in slope (or plateau) as exercise progresses [77–81]

${\dot{V}}_{{\text{O}}_{2}}$ efficiency slope (OUES)	(i) Functional impairment and prognosis in CHF [18, 89–94]	Mortality in CHF <1.05 L/min/log (L/min) or <65% predicted [89]
	(ii) Response to interventions in CHF [95]	
	(iii) More sensitive to training than the $\Delta \;{\dot{V}}_{\text{E}}$/$\Delta {\dot{V}}_{{\text{CO}}_{2}}$ slope in CHF [96]	

${\dot{V}}_{{\text{O}}_{2}}$ efficiency plateau (OUEP)	Functional impairment and prognosis in CHF [89]	Mortality in CHF <25 mL/L or <65% predicted [89]

Ventilatory		

Excess exercise ventilation	(i) Prognosis in PAH [97, 98] and CHF, even under *β*-blocker therapy (CHF) [99, 100]	<age—and gender-specific lower limits of normality [10, 11]
	(ii) Responsive to therapy in CHF [101–103], PAH [104, 105], and CTEPH [106]	
	(iii) Responsive to exercise training [107]	Mortality in CHF
		$\Delta {\dot{V}}_{\text{E}}$/$\Delta {\dot{V}}_{{\text{CO}}_{2}(\text{rest-RCP})}$≥ 34 [108] $\Delta {\dot{V}}_{\text{E}}/\Delta {\dot{V}}_{{\text{CO}}_{2}(\text{rest-PEAK})}$≥ 45 [109]
		
		Mortality in PAH
		${\dot{V}}_{\text{E}}$/$\Delta {\dot{V}}_{{\text{CO}}_{2}\text{nadir}}$ ≥ 52 [97]
		${\dot{V}}_{\text{E}}/{\dot{V}}_{{\text{CO}}_{2}}$LT ≥ 54 [98]
		$\Delta {\dot{V}}_{\text{E}}$/$\Delta {\dot{V}}_{{\text{CO}}_{2}(\text{rest-RCP})}$ ≥ 62 [98]
		$\Delta {\dot{V}}_{\text{E}}$/$\Delta {\dot{V}}_{{\text{CO}}_{2}(\text{rest-PEAK})}$ ≥ 48 [97]
		
		Postoperative complications of lung resection
		$\Delta {\dot{V}}_{\text{E}}$/$\Delta {\dot{V}}_{{\text{CO}}_{2}(\text{rest-RCP})}$ ≥ 34 [110]

End-tidal partial pressure for CO_2_ (*P*_ET_CO_2_)	(i) Adjunct for the diagnosis of PVD [111]	Diagnosis of PVD [111]
	(ii) Prognosis in CHF [112–116]	“likely” = ≤ 30 mmHg at the LT
	(iii) Marker of disease severity in PAH [97, 111, 117, 118]	“very likely” = ≤ 20 mmHg at the LT
	(iv) Diagnosis of a patent forame ovale in PAH [119]	progressive reductions as exercise increases
	(v) Responsive to drug therapy in PAH[105] and CHF [101]	sudden increase with exercise cessation
	(vi) Responsive to exercise training [120]	
		Mortality in CHF
		≤33 mmHg at rest [112, 114]
		≤36 mmHg at the LT [115]
		<31 mmHg at peak [116]

Exertional oscillatory ventilation	(i) Indicative of worsening clinical status, severe hemodynamic dysfunction, and reduced functional capacity in CHF [121–126]	Three or more regular ${\dot{V}}_{\text{E}}$ oscillations (standard deviation of three consecutive cycle lengths within 20% of their average), with minimal average amplitude of ventilatory oscillation of 5 L/min [27]
	(ii) Responsive to interventions in CHF [101]	

Cardiovascular		

ΔHeart rate$/\Delta \dot{V}$O_2_ (beat/L)	(i) Indicative of abnormal cardiovascular response to exercise [127–130]	<age—and gender-specific lower limits of normality [9, 10]
	(ii) Adjunct for the diagnosis of myocardial ischemia [88, 131–133]	Changes in linearity with increases in steepness [88, 132, 133]

Heart rate recovery (HRR) (beats/min)	(i) Prognosis in asymptomatic subjects referred for exercise testing [134], CHF [135], PAH [28], Type 2 diabetes [136], and COPD [137]	Mortality in patients referred for exercise testing
	(ii) Disease severity in metabolic syndrome [138], obstructive sleep apnea [139], sarcoidosis [140], rheumatological diseases [141, 142], polycystic ovary syndrome [143], polycystic kidney disease [144], and HIV infection [145]	Treadmill, cooldown: HRR_1__min_ ≤ 12 [134, 150, 151]
	(iii) Responsive to aerobic training in CHF, COPD, obstructive sleep apnea, and systemic lupus erythematosus [146–149]	Treadmill, no cooldown: HRR_1__min_ ≤ 18 [135] HRR_2__min_ ≤ 22 [152]
		
		Treadmill, no cooldown: HRR_2__min_ ≤ 42 [153]
		
		
		Mortality in CHF
		Treadmill, cooldown:
		HRR_1__min_ < 6.5 [154]
		
		Treadmill, no cooldown: HRR_1__min_ ≤ 12 [155]
		
		Bike, cooldown: HRR_1__min_ < 17 [156]
		
		Mortality in PAH Bike, cooldown: HRR_1__min_ ≤ 18 [28]
		
		Mortality in COPD Bike, cooldown: HRR_1__min_ ≤ 14 [137]
		Mortality in Type 2 diabetes
		Treadmill, cooldown:
		HRR_1__min_ < 12 HRR_2__min_ < 28 [136]

${\dot{V}}_{{\text{O}}_{2}}$: oxygen uptake; ${\dot{V}}_{{\text{CO}}_{2}}$: carbon dioxide output; ${\dot{V}}_{\text{E}}$: minute ventilation; COPD: chronic obstructive pulmonary disease; CHF: chronic heart failure; PAH: pulmonary arterial hypertension; PVD: pulmonary vascular disease; RCP: respiratory compensation point.

## Abbreviations

CAD: Coronary artery disease
CHF: Chronic heart failure
COPD: Chronic obstructive pulmonary disease
CPET: Cardiopulmonary exercise testing
CTEPH: Chronic thromboembolic pulmonary hypertension
EOV: Exertional oscillatory ventilation
FEV_1_: Forced expiratory volume in one second
FVC: Forced vital capacity
GET: Gas exchange threshold
HR: Heart rate
HRR: Heart rate recovery
LA: Lactic acid
LT: Lactate threshold
OUES: Oxygen uptake efficiency slope
OUEP: Oxygen uptake efficiency plateau
PAH: Pulmonary arterial hypertension
*P*_a_: Arterial partial pressure
*P*_A_: Alveolar pressure
*P*_ET_: End-tidal partial pressure
PVD: Pulmonary vascular disease
R: Respiratory exchange ratio
RCP: Respiratory compensation point
SpO_2_: Pulse oxygen saturation
Unl: Unloaded pedaling
${\dot{V}}_{{\text{CO}}_{2}}$: Carbon dioxide output
*V*_D_/*V*_T_: Dead space to tidal volume ratio
${\dot{V}}_{\text{A}}$: Alveolar ventilation
${\dot{V}}_{\text{E}}$: Minute ventilation
${\dot{V}}_{\text{E}}/{\dot{V}}_{{\text{O}}_{2}}$: Ventilatory equivalent for O_2_
${\dot{V}}_{\text{E}}/{\dot{V}}_{{\text{CO}}_{2}}$: Ventilatory equivalent for CO_2_
${\dot{V}}_{{\text{O}}_{2}}$: Oxygen uptake
VT: Ventilatory threshold
WR: Work rate.
